# Spectral–temporal biphoton waveform of photon pairs from cascade-type warm atoms

**DOI:** 10.1038/s41598-020-73610-2

**Published:** 2020-10-02

**Authors:** Jiho Park, Taek Jeong, Han Seb Moon

**Affiliations:** Department of Physics, Pusan National University, Geumjeong-Gu, Busan, 46241 South Korea

**Keywords:** Quantum optics, Single photons and quantum effects

## Abstract

We investigate the spectral–temporal biphoton waveforms of the photon pairs emitted from cascade-type two-photon-coherent warm ^87^Rb atoms via the spontaneous four-wave mixing process in the 5*S*_1/2_–5*P*_3/2_–5*D*_5/2_ transition, under the condition of the different detuning frequencies (symmetric detuning conditions of ± 1 GHz) of the pump and coupling lasers relative to the 5*P*_3/2_ state. In both detuning cases corresponding to ± 1 GHz, the biphoton temporal waveforms and biphoton spectral waveforms of the photon pairs are measured by means of time-resolved coincidence photon counting and stimulated measurements, respectively. Although photon-pairs were generated using opposite detunings, we confirm that the spectral–temporal biphoton waveforms of the photon pairs are very similar. Furthermore, we observe Hong–Ou–Mandel interference with 82% visibility with the two independent heralded single photons.

## Introduction

One of the advantages of photon pairs generated from atomic ensembles is the indistinguishability of the pairs^1–16^. The properties of the photon source are important for generating interfering photons from a series independent photon pairs^17,18^. Photon-pair sources generated from cold and warm atomic ensembles have been experimentally demonstrated via the spontaneous four-wave mixing (SpFWM) process^1–18^. Recently, highly bright, entangled SpFWM photon-pair sources from a warm atomic ensemble have been demonstrated by exploiting the collective two-photon coherence in a cascade-type atomic system^16^. In such a case, the frequency–time entanglement of the photon pair is very strong, and further, the temporal purity of the heralded single photon is high due to the longer coherence time of the emitted photons relative to the resolution time of the detectors^18–20^. In particular, the merits of brightness, stability, and temporal purity of the generated pairs have attracted research interest with regard to applications involving high-performance correlated photon-pairs, for example, time-energy entangled photon pairs, two-photon interferences of nondegenerate photon pairs, polarization-entangled photons, and high-visibility Franson interference of time-energy entangled photon pairs^20–23^.

Photon pairs generated via the cascade-type spontaneous process from an atomic medium are strongly correlated due to the collective two-photon coherence of the Doppler-broadened atomic ensemble with the two-photon resonant interaction of the pump and coupling lasers^20^. The cascade-type SpFWM process consists of one cascade scheme between the pump and coupling lasers and another cascade scheme between the signal and idler photon pair^13–16^. Although the characterization of frequency–time-entangled photon pairs in cascade-type atomic ensembles has been investigated recently^20^, the purity of the heralded single photon and spectral–temporal biphoton waveforms of the photon pairs according to the detuning frequencies of the pump and coupling lasers in a cascade-type atomic system have never been reported, because it is difficult to measure the spectral biphoton waveform of narrowband entangled photon pairs from atomic ensembles.

In this work, we investigate the spectral–temporal biphoton waveforms of photon pairs generated in the 5*S*_1/2_–5*P*_3/2_–5*D*_5/2_ transition of ^87^Rb atoms for two cases: the detuning frequencies of + 1 GHz and − 1 GHz from the one-photon transitions of the pump laser under the cascade-type two-photon resonant condition. The biphoton temporal waveform (BTW) and biphoton spectral waveform (BSW) of the photon pairs are measured by time-resolved coincidence photon counting and stimulated measurements, respectively. Also, the BSW of the photon pairs is numerically calculated considering the optical depth of the atomic medium, phase-matching condition, and nonlinear coefficient for the SpFWM process using a Doppler-broadened four-level atomic model. In addition, we experimentally demonstrate Hong–Ou–Mandel (HOM) interference between two independent heralded single photons on the different detuning frequencies of the pump and coupling lasers relative to the 5*P*_3/2_ state.

## Experimental configuration

**Figure 1:**
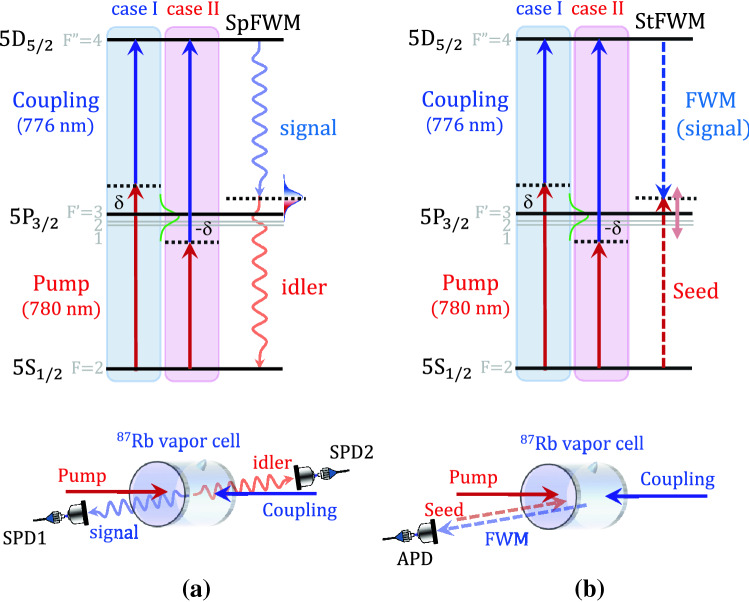
Experimental configuration for spontaneous four-wave mixing (SpFWM) and stimulated FWM (StFWM) generation of 5*S*_1/2_–5*P*_3/2_–5*D*_5/2_ transition of ^87^Rb atoms. (**a**) SpFWM with counter-propagating pump and coupling lasers in ^87^Rb atomic vapor cell. (**b**) StFWM process using seed laser depicting the two cases of the symmetric detuning condition of δ =  + 1 GHz (case I) and − 1 GHz (case II).

Figure 1a shows the schematic for the generation of frequency–time-entangled photon pairs from a cascade-type atomic medium coherently interacting with pump and coupling fields. In our experiment, the 5*S*_1/2_–5*P*_3/2_–5*D*_5/2_ transition of ^87^Rb was used as the cascade-type atomic system, which consists of a 5*S*_1/2_ ground state, 5*P*_3/2_ first excited state, and 5*D*_5/2_ s excited state. The total frequency of the pump and coupling lasers satisfied the conditions for two-photon resonance for the 5*S*_1/2_–5*P*_3/2_–5*D*_5/2_ transition.

For a high-quality photon-pair source from an atomic ensemble, the detuning frequency δ = δ_p_ = − δ_c_ from the one-photon transitions (detuning frequency δ_p_ from the 5*S*_1/2_–5*P*_3/2_ transition for the pump laser and detuning frequency δ_c_ from the 5*P*_3/2_–5*D*_5/2_ transition for the coupling laser) is important for reducing non-correlated photons due to one-photon resonance. In photon-pair generation from the cascade-type warm atomic medium, the two-photon resonance condition (δ_p_ = − δ_c_) should be satisfied because the SpFWM process is very strongly correlated to two-photon coherence.

To investigate the dependence of the detuning frequency δ on the properties of the generated photon pair, we considered the photon pairs in the two cases of the symmetric detuning conditions of δ =  + 1 GHz (case I) and − 1 GHz (case II), as shown in Fig. 1a. The signal and idler photons are emitted in the 5P_3/2_–5D_5/2_ and 5S_1/2_–5P_3/2_ transitions, respectively, under the phase-matching conditions of the pump and coupling lasers. In our experiment, the two-photon resonance condition with the counter-propagating pump and coupling lasers is satisfied by the 5S_1/2_–5D_5/2_ transition of ^87^Rb atoms^16,18^. The Doppler-broadened atoms can be coherently two-photon-excited owing to the Doppler-free two-photon resonant configuration. In our study, the pump and coupling laser powers were set to 0.5 and 10 mW, respectively, with the beam diameter being 1.2 mm, and the polarizations of both lasers are linear and perpendicular to each other. The atomic vapor cell used for our experiment was 12.5 mm in length and contained enriched ^87^Rb atoms (enrichment level of > 98%). The vapor cell temperature was stabilized at 52 °C. In order to remove uncorrelated fluorescence, we used interference filters with a 3-nm bandwidth and etalon filters with 0.95-GHz bandwidth. The emitted signal and idler photons were coupled into two single-mode fibers positioned at an angle of 1.3° relative to the counter-propagating pump and coupling lasers.

Here, we note that we can directly measure the BTW of the narrowband photon-pair via time-resolved coincidence photon counting using two single-photon detectors (SPDs) (PerkinElmer SPCM-AQRH-13HC)^16^. To measure the BSW of the narrowband photon-pairs from an atomic ensemble using the stimulated method via the stimulated FWM (StFWM) process^24^, the seed-laser frequency was scanned around the idler-photon mode corresponding to the 5*S*_1/2_–5*P*_3/2_ transition, as shown in Fig. 1b. The seed laser was aligned to the idler-photon mode. The FWM light from the warm atomic medium was stimulated by the 0.1-mW weak seed laser under the condition of two-photon resonance and detected by an avalanche photodiode (APD) in the phase-matched direction. The seed laser was linearly polarized perpendicular to the polarization of the pump laser.

## Results

The measurement of spectral–temporal biphoton waveforms is important for the characterization of frequency–time-entangled photon pairs. As regards narrowband photon pairs from atomic ensembles, their properties are easy to measure in time but difficult to spectrally resolve because of the long coherence time and narrow spectral bandwidth of the photons.

### Temporal biphoton waveforms

Meanwhile, we can easily measure the normalized second-order cross-correlation function G_*SI*_^(2)^(*τ*) between the signal and idler photons obtained via measuring the coincident detection histogram as a function of τ, where τ denotes the detection time difference (*τ* = *t*_*i*_ − *t*_*s*_) between the idler and signal photons. The G_*SI*_^(2)^(*τ*) is proportional to $\left| {\left\langle 0 \right|a_{s}^{\dag } (t_{s} )a_{i}^{\dag } (t_{i} )\left| {\Psi \left( {t_{s} ,\,\,t_{i} } \right)} \right\rangle } \right|^{2}$, where $\hat{a}_{s}^{\dag } \left( {t_{s} } \right)$ and $\hat{a}_{i}^{\dag } \left( {t_{i} } \right)$ denote the creation operators for the signal and idler photons, respectively, $\left| {\Psi \left( {t_{s} ,\,\,t_{i} } \right)} \right\rangle$ represents the quantum state of the photon pair in the time domain.

**Figure 2:**
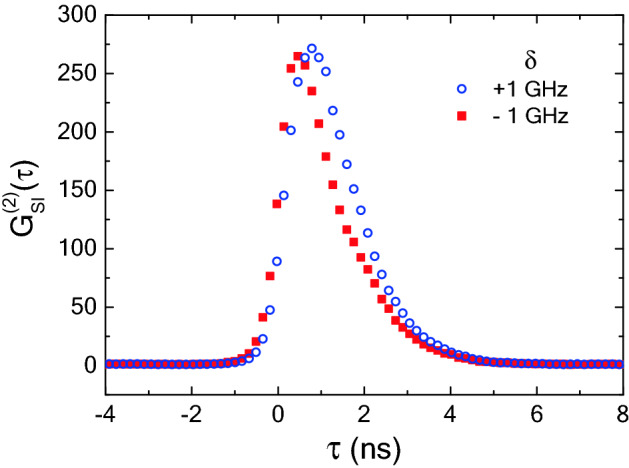
Normalized second-order cross-correlation function G_*SI*_^(2)^(*τ*) between signal and idler photons obtained via spontaneous four-wave mixing (SpFWM) process in Doppler-broadened atomic ensemble; the blue circles and red squares of the G_*SI*_^(2)^(*τ*) spectra represent the two cases of symmetric detuning conditions of δ =  + 1 GHz (case I) and − 1 GHz (case II). We obtained G_*SI*_^(2)^(*τ*) between the signal and idler photons by normalizing the measured coincidence counts during 3-min to the accidental coincidence counts.

Figure 2 shows the G_*SI*_^(2)^(*τ*) plot of the photon pair emitted from the atomic medium, obtained by time-resolved coincidence measurements with a time-correlated single-photon counting (TCSPC) module (PicoQuant PicoHarp 300). The blue circles and red squares in Fig. 2 denote the G_*SI*_^(2)^(*τ*) spectra corresponding to the two cases of symmetric detuning conditions of δ =  + 1 GHz (case I) and − 1 GHz (case II), respectively. The maximum values of G_*SI*_^(2)^(*τ*) are measured to be 271 for δ =  + 1 GHz and 265 for δ =  − 1 GHz. The Cauchy–Schwarz inequality is violated by a factor of more than 10,000. Thus, we can confirm a strong time correlation between the paired photons in both cases. However, the temporal shape and spectral width of the G_*SI*_^(2)^(*τ*) spectra are nearly overlapped but slightly different. The full-width at half-maximum (FWHM) values of the G_*SI*_^(2)^(*τ*) curves are measured to be 1.78 ns for δ =  + 1 GHz and 1.43 ns for δ =  − 1 GHz. From this difference, we note that the coherence time of the photons for δ =  + 1 GHz is shorter than that for δ =  − 1 GHz^18^.

### Spectral biphoton waveforms

As is well-known, the BTW and BSW of narrowband continuous wave (CW)-mode photon pairs exhibit the Fourier-transformed time–frequency relationship. However, although the BSW of the photon pair may be estimated from the measured BTW shown in Fig. 2, it is difficult to obtain information about the absolute optical frequency of the BSW. In this regard, recently, the stimulated measurement of the BSW of photon pairs emitted from an atomic ensemble has been experimentally demonstrated with megahertz-level spectral resolution^24^.

Here, we briefly describe the stimulated measurement of the BSW of the narrowband photon pair using the weak seed laser (Fig. 1b). Owing to the close connection between the SpFWM and StFWM processes^25,26^, the number of photon pairs emitted in the SpFWM process is proportional to the number of the photons induced in the StFWM process. We can measure the power spectrum P(ω) of the generated StFWM field. Here, we note that P(ω) is proportional to $\left| {\left\langle 0 \right|a_{s}^{\dag } (\omega_{s} )a_{i}^{\dag } (\omega_{i} )\left| {\Psi \left( {\omega_{s} ,\,\,\omega_{i} } \right)} \right\rangle } \right|^{2}$, where $\left| {\Psi \left( {\omega_{s} ,\,\,\omega_{i} } \right)} \right\rangle$ represents the quantum state of the SpFWM photon pair in the frequency domain. Therefore, the stimulated method allows us to measure the BSW of the photon pair with the absolute optical frequency information.

**Figure 3:**
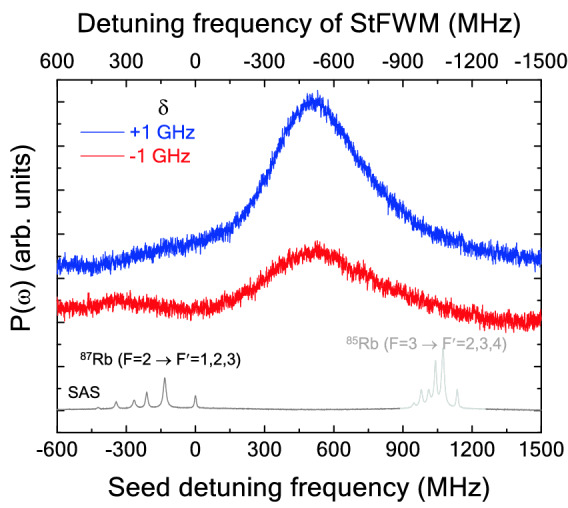
Power spectrum *P*(*ω*) of photon pair in Doppler-broadened atomic ensemble obtained via stimulated measurement. The frequency scales of the bottom and top x-axes represent the detuning frequency of the idler mode from the absolute frequency (384 228 115 MHz) of the 5S_1/2_(F = 2) − 5P_3/2_(F′ = 3) transition and that of the signal mode from the absolute frequency (386 341 017 MHz) of the 5P_3/2_(F′ = 3) − 5D_5/2_(F″ = 4) transition of ^87^Rb, respectively. The scales of the y-axis for the two curves are same and there is an arbitrary shift between the two curves for easy distinction between both curves. Actually, the zero value of the y-axis is that at the detuning frequency of − 600 MHz. The blue and red curves indicate the *P*(*ω*) spectra in the two cases of the symmetric detuning conditions of δ =  + 1 GHz and − 1 GHz, respectively. The gray curve denotes the saturated absorption spectrum (SAS) of the seed laser.

Figure 3 shows the *P*(*ω*) spectra as a function of the seed-laser detuning frequency from the 5S_1/2_(F = 2) − 5P_3/2_(F′ = 3) resonance in the two cases of symmetric detuning of δ =  + 1 GHz (blue curve) and − 1 GHz (red curve), wherein the gray curve indicates the saturated absorption spectrum (SAS) of the seed laser. We can determine the absolute optical frequency of the *P*(*ω*) spectra owing to the SAS of the D_2_ transition of ^87^Rb atoms. However, because the optical frequency of the emitted StFWM signal corresponds to that of the signal photon, the frequency scale of the StFWM signal is expressed in the top x-axis of Fig. 3 as the detuning frequency from the absolute frequency of the 5P_3/2_(F′ = 3) − 5D_5/2_(F″ = 4) transition of ^87^Rb.

The center frequency of the *P*(*ω*) spectra in Fig. 3 is shifted by approximately + 510 MHz from the 5S_1/2_(F = 2) − 5P_3/2_(F′ = 3) transition of ^87^Rb. The key cause underlying this center frequency shift of the *P*(*ω*) spectra is the absorption and dispersion of the atomic medium^24^. However, regardless of the opposite detuning frequency conditions of δ =  + 1 GHz and − 1 GHz, it is interesting to note that the center frequency shift of the *P*(*ω*) spectrum is the same in both cases. From this result, we can confirm that the spectral shape of the photon pairs is not significantly changed in both cases of the symmetric detuning condition. Here, the FWHM values of the *P*(*ω*) spectra are measured to be 421 MHz for δ =  + 1 GHz and 539 MHz for δ =  − 1 GHz. Considering the Fourier transform relationship, the FWHM difference of the *P*(*ω*) spectra in the frequency-domain corresponds to that of the G_*SI*_^(2)^(*τ*) curves of Fig. 2 in the time domain.

However, the magnitude of the *P*(*ω*) spectrum for δ =  + 1 GHz is estimated to be 2.3 times larger than that for δ =  − 1 GHz. The magnitude difference of the *P*(*ω*) spectra emerges as the difference of the coincidence counting rates of the photon pairs. Under the same experimental conditions, the coincidence counting rates are measured to be 5500 counts/s for δ =  + 1 GHz and 2.4 kHz for δ =  − 1 GHz with the coincidence window of 6 ns.

### Numerical calculation of spectral biphoton waveforms

To elucidate the spectral property of the photon pair according to the detuning condition of δ, we calculated *P*(*ω*) of the SpFWM biphoton state $\left| {\Psi \left( {\omega_{s} ,\,\,\omega_{i} } \right)} \right\rangle$ from a simplified four-level cascade-type atomic system^14,25^. Considering the optical depth (OD) of a warm atomic medium, *P*(*ω*) can be written as

1 $$P(\omega ) = \left| {\alpha (\omega )\,\varphi (\omega )\,\kappa (\omega )} \right|^{2} ,$$

where $\alpha \left(\omega \right)$,$\varphi \left(\omega \right)$, and $\left(\omega \right)$ represent the power profile of the signal and idler photons in the warm atomic medium, longitudinal phase-matching function, and nonlinear parametric coupling coefficient corresponding to the SpFWM process, respectively. Further, $\omega$ denotes the frequency detuning from the central frequency of the signal or idler photons. When the pump and coupling lasers are applied to a warm atomic ensemble of ^87^Rb atoms of length *L*, $\alpha \left(\omega \right)$,$\varphi \left(\omega \right)$, and $\left(\omega \right)$ can be, respectively, expressed as

2 $$\alpha (\omega ) = e^{{ - i(k_{s} + k_{i} )L/2}} ,$$

3 $$\varphi (\omega ) = {\text{sinc}}\left( {\frac{\Delta kL}{2}} \right),$$

4 $$\kappa (\omega ) = \frac{{\sqrt {\overline{\omega }_{s} \,\overline{\omega }_{i} } }}{2ic}\chi^{(3)} (\omega )E_{p} E_{c} ,$$

where Δ*k* denotes the wave-vector mismatch of the four fields, i.e., $\Delta k = k_{p} + k_{c} - k_{s} - k_{i}$, with ${k}_{p,c,s,i}$ denoting the wave vectors, respectively, of the pump, coupling, signal, and idler fields; ${\overline{\omega }}_{s}$ and ${\overline{\omega }}_{i}$ the central frequencies of the signal and idler photons, respectively; ${}^{\left(3\right)}(\omega$) the third-order susceptibility of the atomic ensemble; *c* the speed of light in vacuum; ${E}_{p}$ and ${E}_{c}$ the amplitudes of the pump and coupling electric fields, respectively.

**Figure 4:**
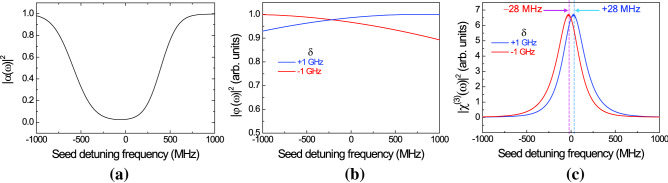
Numerically calculated (**a**) ${\left|\alpha (\omega )\right|}^{2}$, (**b**)${\left|(\omega )\right|}^{2}$, and (**c**) ${\left|{}^{\left(3\right)}(\omega )\right|}^{2}$ in spontaneous four-wave mixing (SpFWM) process using Doppler-broadened four-level atomic model; the blue and red curves correspond to the two cases of symmetric detuning conditions of δ =  + 1 GHz and − 1 GHz, respectively.

Figure 4 shows the calculated results of ${\left|\alpha (\omega )\right|}^{2}$, ${\left|(\omega )\right|}^{2}$, ${\left|{}^{\left(3\right)}(\omega )\right|}^{2}$, respectively. In Fig. 4a, the calculated ${\left|\alpha (\omega )\right|}^{2}$ considers the OD of the 12.5-mm-long atomic ensemble at the vapor cell temperature of 52 °C. The OD value forms an important experimental parameter factor in correlated-photon-pair generation. The spectral feature of ${\left|\alpha (\omega )\right|}^{2}$ corresponds to the absorption spectrum of the idler mode of the 5S_1/2_–5P_3/2_ transition of ^87^Rb atoms. The spectral property of the photon pair generated via SpFWM is strongly modified due to that of ${\left|\alpha (\omega )\right|}^{2}$, but independent of the detuning frequency δ of the pump and coupling lasers.

In our experiment, the signal and idler photons were generated in the phase-matched direction and collected in two single-mode fibers aligned at 1.3° relative to the pump and coupling lasers. Here, we note that the longitudinal phase-matching function ${\left|(\omega )\right|}^{2}$ of Fig. 4b is related to the angle of the generated photon pair. From Fig. 4b, we note that the calculated ${\left|(\omega )\right|}^{2}$ as a function of the tilt angle is different for δ =  + 1 GHz (blue curve) and − 1 GHz (red curve). In the positive range of the seed detuning frequency, the relative magnitude of ${\left|(\omega )\right|}^{2}$ at δ =  + 1 GHz is slightly larger than that at δ =  − 1 GHz.

However, the key term of the calculated *P*(*ω*) via the SpFWM process from the cascade-type atomic ensemble is the third-order susceptibility ${}^{\left(3\right)}(\omega$) in Eq. (4). Figure 4c shows the calculated ${\left|{}^{\left(3\right)}(\omega )\right|}^{2}$ at δ =  + 1 GHz (blue curve) and − 1 GHz (red curve) as a function of the frequency of the seed laser. We note that there is a center frequency shift of + 28 MHz for δ =  + 1 GHz and − 28 MHz for δ =  − 1 GHz. The center frequency shift of ${}^{\left(3\right)}(\omega$) is why the nonlinear effect of ${}^{\left(3\right)}(\omega$) is only weakly influenced by the three-photon coherence including the cascade-type two-photon coherence with the pump and coupling lasers and the V-type two-photon coherence with the pump and seed lasers in the Doppler-broadened profile. As δ increases, the magnitude of ${}^{\left(3\right)}(\omega$) significantly decreases. Therefore, in the SpFWM process, the spectral feature of the photon pair is influenced by the detuning frequencies of the pump and coupling lasers.

**Figure 5:**
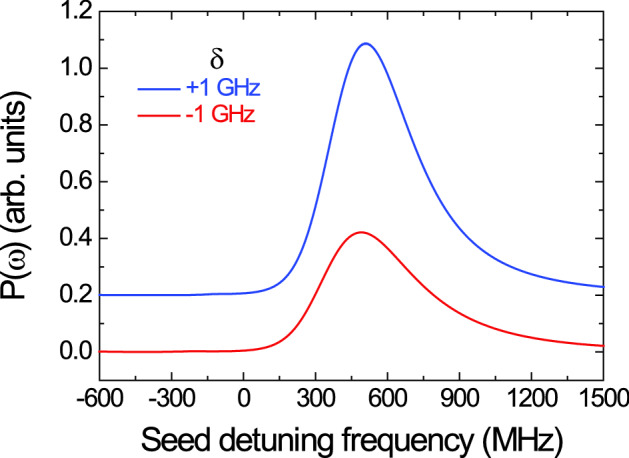
Numerically calculated *P*(*ω*) spectra in two cases of symmetric detuning conditions of δ =  + 1 GHz (blue curve) and − 1 GHz (red curve) using Doppler-broadened four-level atomic model.

Figure 5 shows the *P*(*ω*) spectra calculated from Eq. (1) in the two cases of symmetric detuning of δ =  + 1 GHz (blue curve) and − 1 GHz (red curve) considering the etalon filter with the 0.95-GHz bandwidth. The theoretical results of Fig. 5 are in partial agreement with the experimental results of Fig. 3. In the *P*(*ω*) spectra calculated as functions of the detuning frequency of the seed laser, this center frequency shift of *P*(*ω*) due to the absorption and dispersion of the atomic medium is clearly apparent. Furthermore, the magnitude of the *P*(*ω*) spectrum for δ =  + 1 GHz is calculated to be approximately 2.3 times larger than that for δ =  − 1 GHz, because of the asymmetry of ${\left|\alpha (\omega )\right|}^{2}$ due to the hyperfine structures of the 5*P*_3/2_ state, the slight difference of ${\left|(\omega )\right|}^{2}$ at the tilt angle of 1.3°, and the center frequency shift of ${\left|{}^{\left(3\right)}(\omega )\right|}^{2}$. However, the calculated *P*(*ω*) spectra of Fig. 5 are not in complete agreement with the observed spectral shapes of Fig. 3 because the hyperfine structures of the 5*P*_3/2_ and 5*D*_5/2_ states are not considered in the simple four-level atomic model. From the theoretical results of Figs. 4 and 5, we can qualitatively elucidate the spectral properties of the photon pair generated from a Doppler-broadened cascade-type atomic ensemble under the symmetric detuning conditions of δ =  + 1 GHz and − 1 GHz.

### Time-resolved Hong–Ou–Mandel interference

**Figure 6:**
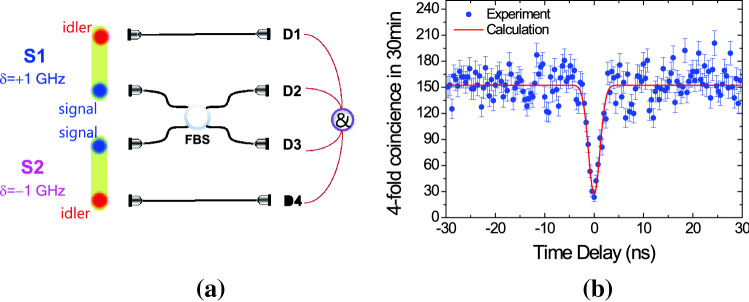
Time-resolved Hong–Ou–Mandel (HOM) interference. (**a**) The schematic of experimental setup for HOM interference between two independent CW photon pairs of S1 (δ =  + 1 GHz) and S2 (δ =  − 1 GHz) from Doppler-broadened atomic ensembles under symmetric detuning conditions (FBS: fiber beam splitter, D: single-photon detector): (**b**) HOM interference between with the two independent heralded single photons in a 30-min effective measurement time interval with a 3-ns temporal window. The red curve represents the theoretical HOM curve.

We measured the HOM interference fringe using two independent heralded single photons of the source1 (S1: δ =  + 1 GHz) and source2 (S2: δ =  − 1 GHz) with the symmetric detuning conditions, as shown in Fig. 6a. The experimental setup and method used to obtain time-resolved HOM interference between two independent narrowband CW-mode photon pairs is the same with that of the previous study^18^. Here, we note that two-source HOM interference is essential to determine the purity of heralded single photons. Figure 6b shows the fourfold coincidence measurement of two independent CW photon pairs in a 30-min effective measurement time interval with a 3-ns temporal window. The *x*-axis represents the arrival time difference (Δ*t*) for the two independent heralded photons, wherein the time step of Δ*t* is set to 0.4 ns. In the study, HOM interference using two independent CW single photons was realized with no synchronization or supplemental filters. Although the two independent photon-pair sources were generated from the atomic ensemble under the different detuning conditions of δ =  + 1 GHz and − 1 GHz, the visibility of HOM interference can be estimated to be 82%. Therefore, we could confirm the identity of independent photon pairs via the cascade-type SpFWM. However, using two independent photon-pair sources photons from identically detuned processes of δ =  + 1 GHz, the HOM interference with the raw visibility of 91.5% can be obtained. The indistinguishability of photons from identically detuned processes is slightly higher than that from two different detuned processes.

## Conclusion

We experimentally investigated the dependence of photon pairs from the 5*S*_1/2_ − 5*P*_3/2_ − 5*D*_5/2_ transition of ^87^Rb atoms on the detuning frequencies δ of the pump and coupling lasers relative to the 5*P*_3/2_ state. In the two cases of the symmetric detuning conditions of δ =  + 1 GHz and − 1 GHz, we measured and compared the properties of the photon pairs in the time and spectral domains. When the BTW of the photon pair was measured by time-resolved coincidence photon counting, the temporal shapes in both cases exhibited a close correspondence with each other, even though the FWHM of the BTW for δ =  + 1 GHz was larger than that for δ =  − 1 GHz. The strong time correlation between the paired photons in both cases was confirmed by the violation of the Cauchy–Schwarz inequality by more than 10,000.

When the BSW of the photon pair was measured by means of a stimulated measurement with a photodiode, we could measure the power spectrum *P*(*ω*) of the photon pair emitted from the atomic ensemble with megahertz-level spectral resolution. In both the cases of δ =  + 1 GHz and − 1 GHz, the center frequencies and spectral shapes of both *P*(*ω*) spectra were similar to each other: the shift was approximately + 510 MHz from the 5S_1/2_(F = 2) − 5P_3/2_(F′ = 3) transition of ^87^Rb. Although the FWHMs and magnitudes of the *P*(*ω*) spectra were different between the two cases, the BSW features of the generated signal and idler photons via the cascade-type SpFWM process did not change according to the different detuning conditions of δ under the two-photon resonant condition.

In addition, to examine the *P*(*ω*) characteristics of the cascade-type SpFWM photon pairs, we theoretically investigated the influence of the optical depth of the atomic medium, the phase-matching condition, and the nonlinear coefficient for SpFWM process using a Doppler-broadened four-level atomic model. The measured *P*(*ω*) spectra were in good agreement with the calculated results. From the calculated results, we confirmed that the absorption and dispersion properties of the atomic ensemble mainly depend on the center frequencies and spectral shapes of the *P*(*ω*) spectra under both detuning conditions. Further, the reason for the magnitude difference of the *P*(*ω*) spectra according to δ can be understood as the difference of the longitudinal phase-matching function and center frequency shift of the third-order susceptibility ${}^{\left(3\right)}(\omega$).

Furthermore, we demonstrated the time-resolved HOM interference with visibility as high as 82%, using two independent photon-pair sources with the detuning frequencies of the pump and coupling lasers in a warm atomic ensemble. Therefore, the identity of independent photon pairs via the cascade-type SpFWM may be relatively robust in comparison with that obtained via the double Λ-type SpFWM process^3–6^. We believe that our results can contribute to a better understanding of the properties of photon pairs obtained via the spontaneous FWM process in a cascade-type atomic medium. Our results can contribute to shedding light on important issues regarding the identity of independent photon pairs for the realization of scalable quantum communication networks.
